# Clinical Efficacy of Hydroxychloroquine in Patients with COVID-19: Findings from an Observational Comparative Study in Saudi Arabia

**DOI:** 10.3390/antibiotics10040365

**Published:** 2021-03-31

**Authors:** Saleh Alghamdi, Bassant Barakat, Ilhem Berrou, Abdulhakim Alzahrani, Abdul Haseeb, Mohamed Anwar Hammad, Sirajudheen Anwar, Abdulmajeed Abdulghani A. Sindi, Hussain A. Almasmoum, Mohammad Albanghali

**Affiliations:** 1 Department of Clinical Pharmacy, Faculty of Clinical Pharmacy, Albaha University, Albaha 57911, Saudi Arabia; saleh.alghamdi@bu.edu.sa (S.A.); bbarakat@bu.edu.sa (B.B.); m.anwar@bu.edu.sa (M.A.H.); 2 School of Health and Social Wellbeing, University of the West of England, Staple Hill, Bristol BS16 1DD, UK; 3 Pharmaceutical Care Services Department, King Fahad Hospital, Albaha 57911, Saudi Arabia; aalzahrani116@moh.gov.sa; 4 Department of Clinical Pharmacy, College of Pharmacy, Umm Al-Qura University, Makkah 24231, Saudi Arabia; amhaseeb@uqu.edu.sa; 5 Department of Pharmacology and Toxicology, College of Pharmacy, University of Hail, Hail 55482, Saudi Arabia; si.anwar@uoh.edu.sa; 6 Department of Basic Medical Science, Faculty of Applied Medical Sciences, Albaha University, Albaha 57911, Saudi Arabia; asindi@bu.edu.sa; 7 Department of Laboratory Medicine, Faculty of Applied Medical Sciences, Umm Al-Qura University, Makkah 24231, Saudi Arabia; Haamasmoum@uqu.edu.sa; 8 Department of Public Health, Faculty of Applied Medical Sciences, Albaha University, Albaha 57911, Saudi Arabia; mohammad.aref@bu.edu.sa

**Keywords:** Hydroxychloroquine, COVID-19, hospital stay, ICU, mechanical ventilation, Saudi Arabia

## Abstract

The aim of this study was to assess the clinical effectiveness of Hydroxychloroquine-based regimens versus standard treatment in patients with the coronavirus disease admitted in 2019 to a hospital in Saudi Arabia. A comparative observational study, using routine hospital data, was carried out in a large tertiary care hospital in Al Baha, Saudi Arabia, providing care to patients with COVID-19 between April 2019 and August 2019. Patients were categorized into two groups: the Hydroxychloroquine (HCQ) group, treated with HCQ in a dose of 400 mg twice daily on the first day, followed by 200 mg twice daily; the non HCQ group, treated with other antiviral or antibacterial treatments according to protocols recommended by the Ministry of Health (MOH) at the time. The primary outcomes were the length of hospital stay, need for admission to the intensive care unit (ICU), time in ICU, and need for mechanical ventilation. Overall survival was also assessed. 568 patients who received HCQ (treatment group) were compared with 207 patients who did not receive HCQ (control group). HCQ did not improve mortality in the treated group (7.7% vs. 7.2%). There were no significant differences in terms of duration of hospitalization, need for and time in ICU, and need for mechanical ventilation among the groups. Our study provides further evidence that HCQ treatment does not reduce mortality rates, length of hospital stay, admission and time in ICU, and need for mechanical ventilation in patients hospitalized with COVID-19.

## 1. Introduction

Coronaviridae viruses have a positive-sense RNA that has an outer viral coat. These viruses cause respiratory infections in humans, manifesting a range of symptoms including those of the common cold and pneumonia [1,2]. The outbreak of severe acute respiratory syndrome (SARS), caused by the SARS corona virus [3,4], in 2003, and the Middle East respiratory syndrome (MERS) outbreak in 2012, slightly increased interest and understanding of the virus [5,6]. However, due to the somewhat confined areas of the outbreak and the minimal disruption to modern life, efforts to tackle infections caused by the family remained limited. In 2019, when the novel Coronavirus (2019-nCoV) was identified in Wuhan in China, causing pneumonia-like symptoms in large populations, serious interest developed since the COVID-19 strain brought modern life to a halt. The virus is believed to have originated in animals before it was transmitted to humans. The virus is highly transmissible among humans through airborne droplets coughed up or sneezed by infected persons. The virus has caused the deaths of hundreds of thousands across the world and continues to be at the top of global concerns [7,8].

COVID-19 infection mostly causes mild to moderate respiratory disease in 80% of infected people. However, about 15% of people may suffer from severe symptoms, and up to 5% of people may get critically ill. The disease can cause death in up to 3% of infected individuals. However, the mortality rate can rise to 15% especially in infected people over the age of 80 [9]. COVID-19 infections, and the strict measures most governments around the world introduced to curb its spread, have had a major economic impact due to closures of workplaces and factories, unemployment, reduced productivity, and increased healthcare costs associated with increased morbidity and mortality [10]. Extensive efforts are ongoing to improve treatment options [11]. There are more than 1087 studies into COVID-19 registered at clinicaltrials.gov, 60% of which are interventional studies [12]. Many are exploring the clinical effectiveness of antimalarial and antiviral agents in improving the clinical outcomes of COVID-19 infections [13]. Repurposing old drugs to solve novel problems is being increasingly explored by Pharma in an effort to reduce development costs and shorten the timescale to get drugs onto the market [14]. 

The two antimalarial drugs, Chloroquine (CQ) and Hydroxychloroquine (HCQ) represent good examples for such practice and they are suggested to have promising potential to improve the clinical outcomes of COVID-19 patients [15,16,17,18]. Their antiviral mechanisms of action are not fully understood, but four theories have been suggested: they can block viral entry, prevent the viral release into the host, reduce the infectivity of the virus, and/or modulate the immune response [19,20,21].

To date, despite the limited evidence, HCQ, sometimes in combination with the macrolide azithromycin (AZ), is given to COVID-19 patients in many countries to improve patient outcomes [16]. The studies supporting the use of HCQ are often small and use different outcomes, which makes comparing evidence of clinical effectiveness challenging.

A study from France and another one from China first claimed benefits of HCQ in patients with COVID-19 [15,22]. Although there was much criticism of the bold conclusions drawn from the French study, HCQ was authorized for use in France to treat COVID-19 patients based on its results. The US Food and Drug Administration (FDA) also authorized its use in patients not taking part in clinical trials [23]. HCQ has many serious adverse drug reactions including prolongation of the QT interval and increasing the risk of ventricular tachyarrhythmias [24,25,26,27], and several studies reported on the safety of HCQ in COVID-19 patients [17,28], including in combination with azithromycin which can also cause QT prolongation [29]. This is particularly significant in patients who are critically unwell, with multiple organ failure and metabolic abnormalities, as severe COVID-19 patients often are [30].

On March 19, 2020, based on the best available scientific evidence, the Saudi Ministry of Health (MOH) issued the first protocol to help standardize the clinical management of confirmed COVID-19 adult patients [31]. In this version, HCQ in a dose of 400 mg every 12 h for 1 day, followed by 200 mg twice a day for 5–7 days was one of the recommended treatment options for mild to moderate and for severe cases of the disease if there were no contraindications. In later versions, June 17, 2020, HCQ was not among the recommended options for severe cases [31].

To our knowledge, only one small retrospective cohort study [32] evaluating the efficacy of HCQ in the Saudi Arabian population has been published. Furthermore, despite the numerous studies addressing the efficacy and safety of HCQ in COVID-19 patients, existing evidence remains inconclusive. Therefore, our study aims to compare the treatment outcomes of Hydroxychloroquine-based regimens versus standard treatment in COVID-19 patients in Saudi Arabia.

## 2. Results

Results from 775 patients admitted during the period of April 2019 to August 2019 were analyzed. Around 73.3% of the patients received HCQ containing regimens, compared with 26.7% of the patients who were prescribed non-HCQ based treatments. The number of patients in the HCQ group was approximately 2.7-fold the number of patients in the other group. Demographic data of the study patients are listed in Table 1. No significant differences were observed between the two groups regarding age, gender, or nationalities.

Forty-five drug regimens were prescribed for patients. In-patients treated with HCQ alone or in combination with ceftriaxone represent the most prescribed regimens (25.9, 24.1% respectively), whereas in the nonHCQ group, a ceftriaxone monotherapy or in combination with azithromycin accounted for 54.2% of the participants in equal distribution (see Table 2 for additional information).

Results representing the difference between HCQ and non HCQ groups have shown no improvement in the clinical outcomes of the HCQ-treated group. HCQ did not improve mortality in the treated group (7.7% vs. 7.2%). HCQ treated patients had a slightly higher duration of hospitalization and time in ICU. Furthermore, slightly more patients who did not receive HCQ based treatment needed mechanical ventilation (Table 3). Single treatment regimens with HCQ alone, ceftriaxone alone, and azithromycin alone show no significant impact on mortality rates and other clinical parameters. Results are shown in Supplementary Table S1.

Table 4 shows the results of the regression analyses after controlling for age and gender. Patients who received HCQ based treatment stayed longer in hospital, were more likely to need ICU and mechanical ventilation, and spent a longer time in ICU. However, there were no significant differences in these outcomes between the two cohorts.

**Figure 1:**
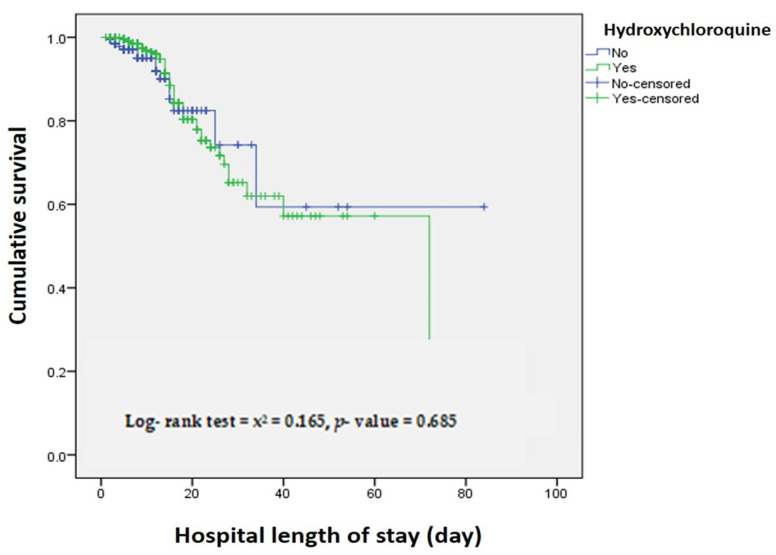
Hospital length of stay (day) by Hydroxychloroquine and non-Hydroxychloroquine treated patients.

When assessing the association between hospital length of stay and survival, the adjusted Cox-regression model shows no significant mortality difference between HCQ and Non-HCQ treated patients (adjusted HR, 1.129 [95% CI, 0.626-2.033], *p*-value = 0.687) (Figure 1 and Table 5).

**Figure 2:**
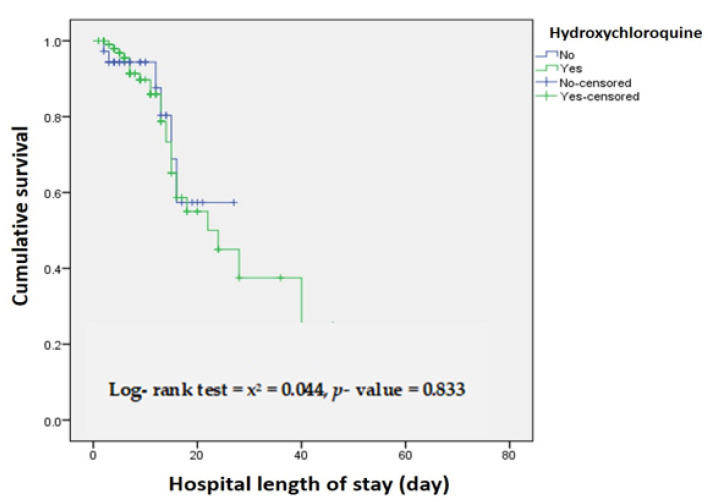
Time in ICU (day) by Hydroxychloroquine and non- Hydroxychloroquine treated patients.

When assessing the association between time in the ICU and survival, the adjusted cox-regression model indicates no significant mortality difference between Hydroxychloroquine and Non-Hydroxychloroquine treated patients (adjusted HR, 0.909 [95% CI, 0.369–2.242], *p*-value = 0.836) (Figure 2 and Table 6).

## 3. Discussion

In this study, we show that treatment with HCQ did not improve clinical outcomes for adults hospitalized for COVID-19 infection, including mortality rate, hospital length of stay, admission to and time in ICU, and need for mechanical ventilation.

Our results are consistent with results from recent observational studies suggesting no antiviral activity for HCQ against SARS-CoV-2. A study in Riyadh by Almazrou et al. found that HCQ treatment did not improve patients’ hospital length of stay and days in ICU [32]. 

A large study in the USA reported that HCQ use among patients hospitalized with COVID-19 did not lower the risk of intubation or death [16]. Recent studies by Mahevas et al. [33] and Rosenberg et al. [17] also showed similar patterns among patients receiving HCQ alone or in combination with AZ. Conclusions from a systematic review [34] point to some benefit of HCQ in relation to radiological progression, time to body temperature normalization, and the number of cough days. However, no impact of HCQ on mortality or reducing the risk of clinical worsening of the disease was reported. Another systematic review and meta-analysis by Mega et al. [35] also confirmed that HCQ does not improve viral clearance, disease worsening, and mortality rates. It rather showed that mortality rates were slightly higher in patients treated with HCQ, several of whom had to stop taking HCQ-based treatment due to severe adverse drug reactions. Our results are in accordance with reports in the literature suggesting that the use of a regimen containing HCQ for treatment of COVID-19 patients did not offer clinical benefits. This lack of benefits could be due to the inability of HCQ to kill the SARS-CoV-2 virus beyond in vitro settings or could be due to the timing of using the medicine late in the disease progression pathway. The latter explanation is based on the possible benefits of HCQ in mildly symptomatic [36] or asymptomatic patients in whom perhaps the suggested mechanism of reducing viral load (and reducing transmission) is not onerous. 

### Strengths and Limitations

To our knowledge, our study is among the few studies that clearly describes treatment option protocols for COVID-19 patients in Saudi Arabia. Research publications related to COVID-19 in Saudi Arabia mostly focused on control and prevention, and on the clinicopathological aspects of the disease [37], and viral genomics and its implication on drug discovery [38]. These studies were mostly narrative, focusing on reported views and experiences. Furthermore, our study included a large sample size, and the evaluated clinical parameters were in accordance with those evaluated in other observational studies, which facilitates comparison.

However, there are some limitations to address. First, although health care in Saudi Arabia has a homogeneous setup, there is some variability in standard protocols among the hospitals that could have led to residual confounding.

Second, the study only included hospitalized adults in Al-Baha province, and findings may not be generalizable to other provinces in the kingdom. 

Third, only one dosing regimen of HCQ was evaluated in the study (a dose of 400 mg twice daily on the first day, followed by 200 mg twice daily).

## 4. Materials and Methods

### 4.1. Study Design and Population

Our study was conducted in King Fahd Hospital (380 beds), the referral hospital for COVID-19 patients in the Al Baha Province (Saudi Arabia). A total of 775 PCR-confirmed COVID-19 patients (age ≥ 18 years) were included in this retrospective hospital-based cohort study. Patients were followed from the time of admission until the time of discharge between April 2019 and August 2019. According to the treatment protocol, patients were categorized into two groups: the HCQ group; treated with HCQ in a dose of 400 mg twice daily on the first day, followed by 200 mg twice daily, and the non HCQ group; treated with other antiviral or antibacterial treatments according to MOH protocols (these include: Ceftriaxone, Azithromycin, Favipiravir, Tamiflu, Ribavirin and Lopinavir/Ritonavir) [31].

### 4.2. Baseline Information Collection

Trained medical personnel collated the information about patients’ demographics, treatment protocols, and outcomes from the patients’ medical records. A checklist was designed and used to record the necessary information from the patients’ medical records.

### 4.3. Outcomes

We explored the impact of HCQ-based treatment on the following outcomes: length of stay in hospital (expressed as the number of days from the patient’s arrival at the hospital until they are discharged), ICU admission, length of time spent in ICU (expressed as the number of days from the day of ICU admission to the day of discharge), the need for mechanical ventilation, and mortality rates.

### 4.4. Statistical Analysis

We used Statistical Package for the Social Sciences software (SPSS; IBM, Armonk, NY, USA, version 20.0) for the analysis. We illustrated descriptive statistics in tables and figures. The Chi-square test and the associated value was reported for association as appropriate. Kaplan–Meier and log-rank tests were used for presenting and comparing hospital length of stay and time in ICU. A *p*-value < 0.05 was considered significant.

### 4.5. Ethics

This study was approved by the Scientific and Research Committee at King Fahad Hospital in Al Baha, Saudi Arabia. The information and data collected were kept confidential. No personal information was included in this study. This is a secondary analysis of anonymized routine surveillance data.

## 5. Conclusions

Our results revealed that the addition of HCQ to COVID-19 treatment protocols did not significantly reduce the length of hospital stay, admission and length of stay in ICU, and need for mechanical ventilation. Our study provides further evidence of the lack of effectiveness of HCQ in treating patients with COVID-19 infection.

## Figures and Tables

**Table 1:** Demographic characteristics of COVID-19 patients.

Characteristic	Total Patients (*n* = 775)		G1 (*n* = 568)		G2 (*n* = 207)		*p*-Value
	*n*	%	*n*	%	*n*	%	
Age							
Less than 30	119	15.4%	84	14.8%	35	16.9%	0.731
30–50	264	34.1%	193	34%	71	34.2%	
<50	392	50.6%	291	51.2%	101	48.8%	
Gender							
Female	368	47.5%	252	44.4%	116	56%	0.004
Male	407	52.5%	316	55.6%	91	44%	
Nationality							
Saudi	640	82.6%	463	81.5%	177	85.5%	0.195
Non-Saudi	135	17.4%	105	18.5%	30	14.5%	

Group 1 (G1): Patient’s treatment regimens include Hydroxychloroquine. Group 2 (G2): Patient’s treatment regimens DO NOT include Hydroxychloroquine.

**Table 2:** Treatment regimens of COVID-19 patients.

Treatment Regimens	Number of Patients	%
Group 1 (*n* = 568)		
Hydroxychloroquine	147	25.9
Hydroxychloroquine + Ceftriaxone	137	24.1
Hydroxychloroquine + Ceftriaxone + azithromycin	76	13.4
Hydroxychloroquine + azithromycin + Tamiflu	50	8.8
Hydroxychloroquine + azithromycin	37	6.5
Hydroxychloroquine + ceftriaxone + favipiravir	21	3.7
Hydroxychloroquine + ceftriaxone + azithromycin + favipiravir	19	3.3
Hydroxychloroquine + ceftriaxone + azithromycin + Tamiflu	18	3.2
Hydroxychloroquine + ceftriaxone + Tamiflu	11	1.9
Hydroxychloroquine + Tamiflu	6	1.1
Hydroxychloroquine + azithromycin + favipiravir	6	1.1
Hydroxychloroquine + azithromycin + Lopinavir/Ritonavir + Tamiflu	5	0.9
Hydroxychloroquine + favipiravir	5	0.9
Hydroxychloroquine + ceftriaxone + azithromycin + favipiravir + Tamiflu	4	0.7
Hydroxychloroquine + ceftriaxone + azithromycin + Lopinavir/Ritonavir + Tamiflu	3	0.5
Hydroxychloroquine + ceftriaxone + Ribavirin + Lopinavir/Ritonavir	3	0.5
Hydroxychloroquine + ceftriaxone + azithromycin + Lopinavir/Ritonavir	3	0.5
Hydroxychloroquine + ceftriaxone + azithromycin + Ribavirin + Lopinavir/Ritonavir	2	0.4
Hydroxychloroquine + ceftriaxone + azithromycin + Ribavirin + Lopinavir/Ritonavir + Tamiflu	2	0.4
Hydroxychloroquine + azithromycin + Ribavirin + Tamiflu	2	0.4
Hydroxychloroquine + Ribavirin	2	0.4
Hydroxychloroquine + azithromycin + favipiravir + Tamiflu	2	0.4
Hydroxychloroquine + ceftriaxone + azithromycin + favipiravir + Tocilizumab + Tamiflu	2	0.4
Hydroxychloroquine + Ribavirin + Lopinavir/Ritonavir + Tamiflu	1	0.2
Hydroxychloroquine + favipiravir + Tocilizumab	1	0.2
Hydroxychloroquine + azithromycin + Lopinavir/Ritonavir	1	0.2
Hydroxychloroquine + ceftriaxone + azithromycin + Tocilizumab	1	0.2
Hydroxychloroquine + ceftriaxone + Ribavirin	1	0.2
Group 2 (*n* = 207)		
Ceftriaxone	56	27.1
Ceftriaxone + azithromycin	56	27.1
Azithromycin	46	22.2
Ceftriaxone + azithromycin + favipiravir	10	4.8
Ceftriaxone + favipiravir	8	3.9
Azithromycin + Tamiflu	7	3.4
Ceftriaxone + Tamiflu	6	2.9
Ceftriaxone + azithromycin + Tamiflu	4	1.9
Favipiravir	4	1.9
Azithromycin + favipiravir	2	1.0
Ribavirin	2	1.0
Ceftriaxone + azithromycin + Tocilizumab	1	0.5
Ceftriaxone + azithromycin + Ribavirin	1	0.5
Ceftriaxone + azithromycin + favipiravir + Tamiflu	1	0.5
Azithromycin + Ribavirin	1	0.5
Tamiflu	1	0.5
Ceftriaxone + Ribavirin + Lopinavir/Ritonavir	1	0.5

**Table 3:** Comparison of outcomes between G1 and G2.

	G1 (*n* = 568)				G2 (*n* = 207)			
Outcome	Mean	SD	Median	IQR	Mean	SD	Median	IQR
Hospital length of stay	14.01	11.74	11	7–17	11.36	9.73	9.5	4–15.7
Time in ICU	11.48	10	9	5-15	9.44	6.32	8	4–13.7
	**N**		**%**		**N**		**%**	
ICU admission	104		18.3%		36		17.4%	
Mechanical ventilator	63		26.8%		21		31.3%	
Mortality rate	44		7.7%		15		7.2%	

SD, standard deviation; IQR, interquartile range.

**Table 4:** Regression analysis results of the outcomes for G1 treatment regimen vs. G2 treatment regimen.

Outcome	Estimates	SE	*p*-Value
Hospital length of stay	0.028	0.04	0.451
ICU admission	−0.063	0.21	0.769
Time in ICU	0.002	0.04	0.972
Mechanical ventilator	−0.599	0.48	0.211
Mortality rate	0.756	0.58	0.191

**Table 5:** Hospital length of stay (day) by Hydroxychloroquine and non- Hydroxychloroquine treated patients.

No of Patients at Risk N (%)				
	Day 1 (Admission)	Day 7	Day 14	Day 28
Hydroxychloroquine	566 (100)	346 (61)	141 (24.9)	31 (5.5)
Non-Hydroxychloroquine	206 (100)	104 (50.5)	41 (20)	7 (3.4)

**Table 6:** Time in ICU (day) by Hydroxychloroquine and non- Hydroxychloroquine treated patients.

No of Patients at Risk N (%)				
	Day 1 (Admission)	Day 7	Day 14	Day 28
Hydroxychloroquine	103 (100)	68 (66)	28 (27)	5 (4.8)
Non-Hydroxychloroquine	35 (100)	19 (54.3)	8 (22.9)	0 (0)

## Data Availability

Data available on request due to ethical restrictions. The anonymized data presented in this study are available on request from the corresponding author. The data are not publicly available to maintain privacy and adhere to guidelines of the ethics protocol.

## Supplementary Materials

The following are available online at https://www.mdpi.com/article/10.3390/antibiotics10040365/s1, Table S1: Comparison of outcomes between HCQ, Ceftriaxone and Azithromycin.
