# Supplementary material for: Clinical Efficacy of Hydroxychloroquine in Patients with COVID-19: Findings from an Observational Comparative Study in Saudi Arabia
Source: Antibiotics (Basel). 2021 Mar 31;10(4):365. doi: 10.3390/antibiotics10040365 (PMC8065820; doi:10.3390/antibiotics10040365)
Supplement: Supplementary file 1 [file antibiotics-10-00365-s001.pdf]

Table S1.: Comparison of outcomes between HCQ, Ceftriaxone and Azithromycin.

	HCQ (n = 147)				Ceftriaxone (n = 56)				p-value HCQ Vs. Ceftriaxone	Azithromycin (n = 46)				p-value HCQ Vs. Azithromycin
**Outcome**	**Mean**	**SD**	**Median**	**IQR**	**Mean**	**SD**	**Median**	**IQR**		**Mean**	**SD**	**Median**	**IQR**	
Hospital length of stay	8.7	8.9	7	3-11	9.2	9.4	6.5	4-11	NS	7	8.4	5	3-8	NS*
Time in ICU	10.4	14.6	7.5	3-13	12	6.4	13	5-18	NS	8.8	9.4	5	3-16	NS
	**N**		**%**		**N**		**%**			**N**		**%**		
ICU admission	22		15		8		14			8		17		
Mechanical ventilator	10		7		4		7			2		4.5		
Mortality rate	6		4		3		5.4			0		0		

SD, standard deviation; IQR, interquartile range; NS, not significant for both comparisons of mean and median; *, significant only for comparison of median with p-value = 0.002.
